# Effect of Sublethal Doses of Imidacloprid on the Biological Performance of Aphid Endoparasitoid *Aphidius gifuensis* (Hymenoptera: Aphidiidae) and Influence on Its Related Gene Expression

**DOI:** 10.3389/fphys.2018.01729

**Published:** 2018-12-11

**Authors:** Zhi-Wei Kang, Fang-Hua Liu, Rui-Ping Pang, Hong-Gang Tian, Tong-Xian Liu

**Affiliations:** ^1^State Key Laboratory of Crop Stress Biology for the Arid Areas, Key Laboratory of Northwest Loess Plateau Crop Pest Management of Ministry of Agriculture, Northwest A&F University, Yangling, China; ^2^Department of Entomology, University of Georgia, Athens, GA, United States; ^3^State Key Laboratory of Integrated Management of Pest and Rodents, Institute of Zoology, Chinese Academy of Sciences, Beijing, China

**Keywords:** *Aphidius gifuensis*, imidacloprid, biological performance, transcriptome, integrated pest management

## Abstract

The integrated pest management (IPM) strategy was developed and used in combination with pesticides and beneficial biological control agents. To further develop IPM efficiency, it is important to evaluate the side effects of pesticides on biological control agents. *Aphidius gifuensis* is one of the most important aphid natural enemies and has been successfully used to control *Myzys persicae* and other aphid species. Imidacloprid (IMD) is a popular pesticide used worldwide and is highly toxic to non-target arthropods. Here, we investigated the short-term sublethal toxicity of IMD in *Aphidius gifuensis* and its impact on the biological performance and gene expression of this parasitoid. We found that sublethal IMD doses had a significant negative effect on the life history traits of female *A. gifuensis*, including shortening the lifespan and lowering parasitic capacity. Moreover, exposure to sublethal IMD also adversely affected the response of *A. gifuensis* to aphid-infested plant volatiles. Based on the transcriptome analysis, we found that the exposure to sublethal IMD doses significantly affected expression of genes involved in the central nervous system, energy metabolism, olfactory, and detoxification system of *A. gifuensis*. RT-qPCR also revealed that short term expose to sublethal IMD doses significantly induced the gene expression of genes related to the central nervous system (*nAChRa7, nAChRa9, TbH, OAR1, NFR, TYR*, and *DAR1*), olfactory system (*OR28* and *IR8a1*), and detoxification system (*CYP49p3, CYP6a2*, and *POD*), while it suppressed the expression of genes involved in the central nervous system (*nAChRa4* and *nAChRb1*), olfactory system (*Orco1, IR8a2*, and *GR1*), and detoxification system (*GST2*). Furthermore, exposure to sublethal doses of IMD also significantly increased the activities of CarEs and POD, whereas we observed no influence on the activities of CAT, GST, and SOD. Our results indicate that sublethal IMD doses might adversely affect the biological performance of *A. gifuensis* by altering gene expression related to the function of olfactory, nervous, energy metabolism, and detoxification systems. Thus, how the use of pesticides directly affect insect population should be considered when used in conjunction with natural pest parasitoids in IPM strategies.

## Introduction

Over the past decade, numerous pesticides have been developed and introduced into agriculture, forestry, horticulture, grain storage, and public/personal health. Around the world, more than 2 million tons of pesticides are used annually (De et al., 2014). About 24.3, 18.2, and 9.7 kg/ha of pesticides were used in 12 villages in six counties in Guangdong, Jiangxi, and Hebei provinces, in China (Zhang et al., 2015)w. The global pesticide cost is estimated to be $81.1 billion by 2021. However, the intensive use of pesticides has posed selective pressure on targeted pest species to develop pesticide resistance or pest resurgence (Desneux et al., 2007; Tabashnik et al., 2009). Over 500 species are resistant to at least one type of pesticide (De et al., 2014). For example, the diamondback moth (*Plutella xylostella*) has developed a resistance to over 91 pesticides, all within 3 years (2015–2017), *Dysdercus koenigii* has developed a very high resistance to acetamiprid (from 33 to 433-fold) and imidacloprid (from 21 to 173-fold) in Punjab, Pakistan (Zhang et al., 2016; Saeed et al., 2018). *P. xylostella* is also resistant to *Bacillus thuringiensis* and its derivatives. This higher resistance of pests lead to the development of novel pesticides and an increase in the quantity and frequency of pesticide application, which not only facilitates the resistance in the target pests but also results in environment contamination. In Thailand, the average pesticide residues found in surface water was 1.3757 ± 0.5014 mg/L (dicrotophos in summer) and 0.3629 ± 0.4338 mg/L (ethion in winter), and the average ethion residues in soil was 42.2893 ± 39.0711 mg/kg (summer), and 90 ± 24.16443 mg/kg (winter) (Harnpicharnchai et al., 2013). The persistent nature of pesticides has entered into various food chains and has bioaccumulated in higher trophic levels inlcuding bees, birds, and mammals (Bayen et al., 2005; Desneux et al., 2007; Kapoor et al., 2011; Dicks, 2013). Thus, to some extent, the adverse effects of pesticides have outweighed the benefits associated with their use.

To minimize chemical pesticides use, various candidate biological control agents have been evaluated, such as the application of trap crop systems, and entomopathogenic fungi, bacteria, predators, and parasitoids (Shah and Pell, 2003; Shelton and Badenes-Perez, 2006; Yang et al., 2009; Walker et al., 2017). For example, blue fluorescent light is widely used in rice paddy fields to control the rice stem borer, *Chilo suppressalis* Walker, and *Tryporyza incertulas* Walker moths (Ishikura, 1950). Alfalfa and mungbean are used as a trap crop in cotton fields for managing lygus bugs, *Lygus Hesperus*, and the mirid *Apolygus lucorum*, respectively (Godfrey and Leigh, 1994; Lu et al., 2009). Two parasitic wasps *Trichogrammatoidea bactrae fumata* Nagaraja and *Trichogrammatoidea cojuangcoi* Nagaraja are successfully applied to control the cocoa pod borer, *Conopomorpha cramerella* Snellen, in the field (Lim and Chong, 1987; Alias et al., 2005). However, these biological control agents such as trap crop systems and commercial inundated releases of parasitoids and predators may not be capable of reducing pest densities to levels that avoid economic losses in a timely manner (Yang et al., 2011). Thus, the proper amalgamation of various control techniques into a unified system may provide a powerful tool to keep pest population levels low and to avoid economic damage. However, amalgamation of various pest control techniques also poses a major challenge: how do we take advantage of each biological technique?

The pesticides that are used in pest management programs must be effective in controlling pests and have a low impact on non-target organisms, such as natural enemies (Desneux et al., 2007; Lu et al., 2009). To determine the residual period of control for an insecticide, is essential to plan insect management strategies, which will influence the spraying frequency and the release time of natural enemies, and in turn, affect the pest control cost. Thus, the residual and toxic effects of pesticides are the most serious bottlenecks in the successful use of pesticides and natural enemies.

Aphids are key insect pests that are responsible for major agricultural losses, particularly because they are vectors of various plant viruses (Van Emden and Harrington, 2017). In the Australian grain industry alone, aphid-related plant injuries, either through direct feeding or virus transfer, represent a potential economic cost of $200–480 million/year (Murray et al., 2013; Valenzuela and Hoffmann, 2015). Current management strategies for broadacre aphids rely primarily on pesticides, either through seed dressings or foliar applications (Dedryver et al., 2010; Chollet et al., 2014). However, due to the strong adaptation and fecundity of aphids, they have developed strong resistance to various pesticides. For example, the green peach aphid (*Myzus persicae*) is resistant to more than 70 different types of synthetic insecticides (Silva et al., 2012).

Imidacloprid (IMD) is one of the most extensively used pesticides in the world (Li et al., 2018). It is sprayed directly onto plants or used as a seed or soil treatment on a number of agricultural products to control a variety of insect pests including plant- and leafhoppers, aphids, termites, whiteflies, and thrips (Li et al., 2018). However, IMD is highly persistent and toxic to non-target animals, including bees (Dicks, 2013). When a bumblebee (*Bombus terrestris*) colony was treated with IMD at a sublethal concentration, it significantly reduced the growth rate and production of queens and workers (Laycock et al., 2012; Whitehorn et al., 2012). In addition, there was a significant decrease in the fecundity of *Orius insidiosus, Orius tristicolor, Hippodamia convergens*, and *Chrysoperla carnea*, which are natural enemies of aphids, when treated with sublethal concentrations of IMD (Mizell and Sconyers, 1992; Sclar et al., 1998; Studebaker and Kring, 2003; Rogers et al., 2007; Funderburk et al., 2013).

*Aphidius gifuensis* Ashmead (Hymenoptera: Braconidae) is one of the most widely distributed and dominant endoparasitoid of pest aphids, including *M. persicae* and *Sitobion avenae* (Fabricius), and are successfully applied in greenhouses for controlling vegetable aphids and in fields for tobacco aphid (*M. persicae*, also known as green peach aphid) management in China (Yang et al., 2009, 2011; Ali et al., 2016; Kang et al., 2017a; Yang F. et al., 2017). Furthermore, Yang et al. (2009) reported that after augmentative releases of *A. gifuensis*, the frequency and quantity of pesticide application could be sustained at a low level for 8 years. However, *A. gifuensis* is sensitive to various agrochemicals (Ohta and Takeda, 2015). For example, after 14 days of exposure to residual permethrin and IMD, also showed high toxicities to *A. gifuensis* (Kobori and Amano, 2004). In this work, we not only evaluated the toxicity of IMD in *A. gifuensis*, but also investigated the biological performance of *A. gifuensis* exposed to sublethal doses of IMD. We hypothesized that sublethal doses of IMD would disrupt parasitoids performance through regulating some genes on the molecular level. Transcriptome technology was applied to explore which of the parasitoid genes could be modulated by IMD and how *A. gifuensis* adjusts its detoxification system to respond to the exposure of IMD.

## Materials and Methods

### Insect Species

*Aphidius gifuensis* used in this work were maintained on *M. persicae*, which was reared on chili pepper (*Capsicum annuum* L., var. “Lingxiudajiao F1”) at 25 ± 1°C with a 16 h light: 8 h dark photoperiod and a relative humidity of 60 ± 5% in an air-conditioned insectary.

### Performance of *A. gifuensis* Exposure to IMD

We used three different dilution magnifications to evaluate the toxicity of IMD on *A. gifuensis*, and distilled water was used as a control. Five plastic vials (length: 8 cm; diameter: 4 cm) were treated with 1 ml IMD or H_2_O. The IMD was swirled inside the vials for 30 s and allowed to air-dry in a hood to simulate the pesticide residues. At the time of exposure, twenty 2-day old *A. gifuensis* female adults were introduced into a vial. Twenty-four hours later, the mortality of *A. gifuensis* was counted and living parasitoids (at least 15) were individually collected to test the effects of IMD on the parasitism, longevity and sex ratio of offspring as described by Kang et al. (2017a) with little modification. Chili pepper plant with 200 second- or third- instar *M. persicae* were placed into a plastic cage (diameter: 13 cm; height: 30 cm) with screen mesh caps. Then, five females of *A. gifuensis* from different treatments (control (CK) or IMD), were introduced into each rearing cage for 8 h. After the parasitism, the aphids and Chili pepper complex was maintained in an incubator. Ten days later, the number of mummified aphids and the sex ratio of all wasps emerging from these mummified aphids were recorded. Five biological replicates were conducted in this work.

To analyze the effect of sublethal doses of IMD on the orientation behavior and gene expression of *A. gifuensis*, the LC_20_ of IMD was used and 24 h later, surviving parasitoids were collected and separated into two groups: one group with thirty living parasitoids was flash-frozen in liquid nitrogen and stored at −80°C for the gene expression analysis; the remaining parasitoids were placed into a PCR tube for orientation behavior. Y-tube olfactometers were used to assess the oriented responses of *A*. *gifuensis* toward healthy and aphid-infested plants. Y-tube was conducted as described by Kang et al. (2018a,b). In total, 100 living parasitoids were tested for the orientation behavior.

### RNA Sequencing

Total RNA was extracted from whole bodies of five female *A. gifuensis* using RNAiso Plus (Takara Bio, Tokyo, Japan), following the manufacturer's instructions. The high quality RNA was used for the further cDNA synthesis and Illumina library generation, which was completed at the Novogene Bioinformatics Technology Co., Ltd. (Beijing, China).

### *De novo* Assembly and Gene Annotation

Transcriptome *de novo* assembly was conducted using a short read assembling program—Trinity with min_kmer_cov set to 2 by default and all other parameters set to default (Grabherr et al., 2011). In order to get comprehensive information about the genes, we aligned the unigenes larger than 150 bp to nr, Nt, KEGG, Swiss-Prot, and COG databases, with e-value < 10^−5^. With nr annotation, we used the Blast2GO program to get GO annotation of unigenes (Conesa et al., 2005). The WEGO software was used next to perform GO functional classification for all unigenes (Ye et al., 2006). The unigene expression levels were calculated by fragments per kb per million reads (FPKM) method, using the formula, FPKM (A) = 10^3^ (10^6^ C)/NL (A: Unigene A; C: number of fragments that uniquely aligned to Unigene A; N: the total number of fragments that uniquely aligned to all Unigenes; L: the base number in the CDS of Unigene A). The FPKM method eliminates the influence of different gene lengths and sequencing levels on the calculation of gene expression; therefore, the calculated gene expression can be directly used for comparing differences in gene expression across samples.

### Expression Analysis

Heat map analysis was performed by the R package of pheatmap (http://www.r-project.org/; R Foundation for Statistical Computing, Wien, Austria). Heatmap plots present the binary log of fold-change of IMD/CK for each gene with a three-color scale (navy, white and firebrick).

RT-qPCR was performed to validate the expression of several genes in *A. gifuensis*. Total RNA was extracted from five whole bodies of 2-day old female *A. gifuensis*, and cDNA was then synthesized from 1 μg total RNA using a PrimeScript® RT reagent Kit with gDNA Eraser (perfect Real Time) (Takara, Tokyo, Japan) according to the manufacturer's protocol. Specific gene primers were designed by Primer Premier 6 (PREMIER Biosoft International, Palo Alto, CA, USA), which are presented in Table S1. In total, three biological replicates, with three technical replicates were conducted, and the qPCR was performed as previously described (Kang et al., 2017b). However, in this study, we used a 2^−ΔCt^ method to evaluate the expression of selected genes (Eakteiman et al., 2018).

### Enzyme Activity Assay

The activities of CarE, SOD, CAT, POD, and GST were measured using commercially available assay kits (Nanjing Jiancheng Bioengineering Institute, Jiangsu, China) as described previously (Kang et al., 2017a).

### Data Analyses

The comparison of performance parameters was subjected to a one-way analysis of variance (ANOVA) followed by the separation of means by the Fisher's protected least significant difference (LSD) test at *P* = 0.05. The gene expression profiles were determined by a student's *t*-test at *P* < 0.05. The orientation behavior of *A. gifuensis* under the different treatments was separated by the Chi-square test (*P* < 0.05). A generalized linear mixed-effects model (GLMM) with a binomial family with the cbind function was then performed to analyze the response to the treatment: yes or no. Except for GLMM, SPSS 22.0 (SPSS Inc., Chicago, IL, USA) was used for the data analyses. GLMMs were performed in the R programming environment (version 3.5.1).

## Results

### Exposure of Sublethal Does of IMD Impaired the Performance of *A. gifuensis*

**Figure 1:**
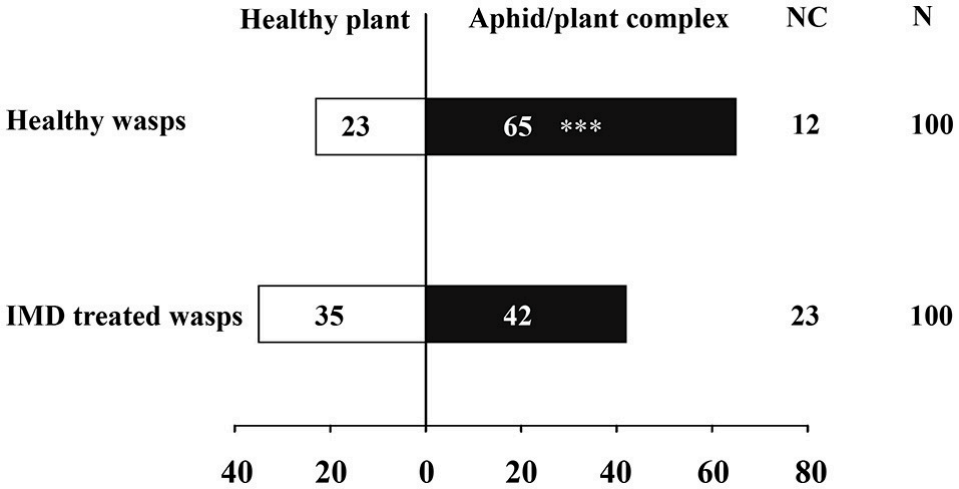
Effects of IMD exposure on the orientation behavior of aphid endoparasitoid, *Aphidius gifuensis* on aphid infested and control plants. Asterisk over the bars designate a significant difference based on GLMM and Chi-square test: ^*^P < 0.05, ^**^P < 0.01, ^***^P < 0.001. *N* = 100.

The influence of IMD on the mortality, parasitism, longevity and female proportion in offspring of *A. gifuensis* are shown in Table 1. Exposure to IMD significantly increased the mortality of female and male adults, and decreased the longevities of surviving female and male adults, as well as the parasitism of surviving female adults (Mortality: Female: *F* = 152.071, *P* < 0.001; Male: *F* = 62.448, *P* < 0.001; Longevity: Female: *F* = 27.952, *P* < 0.001; Male: *F* = 26.069, *P* < 0.001; Parasitism: *F* = 19.991, *P* < 0.001). However, exposure to IMD did not influence the female proportion of offspring produced by surviving female adults, compared to healthy female adults (*F* = 0.725, *P* < 0.504). Furthermore, IMD significantly reduced the sensitivity of *A. gifuensis* to the volatiles from aphid infested plants (Healthy wasps: χ^2^ = 20.045, *P* < 0.001; IMD treated wasps: χ^2^ = 0.636, *P* = 0.425, Figure 1). The GLMM analysis also revealed that IMD changed the response of *A. gifuensis* to these volatiles (*P* = 0.009).

**Table 1:** The side effects of IMD on the parasitism, longevity, and female proportion in offspring of *A. gifuensis*.

**Treatment^a^**	**Survival rate (%)**		**Parasitism (%)**	**Longevity/day**		**Female proportion in offspring (%)**
	**Female**	**Male**		**Female**	**Male**	
CK	100.00 ± 0.00a	99.00 ± 0.78a	70.40 ± 2.50a	12.73 ± 0.51a	11.07 ± 0.57a	59.20a
2,000	0	0	–	–	–	–
4,000	40.00 ± 3.54c	43.00 ± 5.39c	29.20 ± 6.67c	6.60 ± 0.59c	5.20 ± 0.50c	56.60a
8,000	83.20 ± 2.44b	79.20 ± 3.02b	45.20 ± 3.73b	9.47 ± 0.63b	7.93 ± 0.64b	55.20a

a *Treatment: dilution magnification*.

*Means followed by different letters within a column indicate significant difference among the treatments (P < 0.05)*.

### An Overview of the Transcriptome

The transcriptome assembly was performed using the Trinity program, with an optimal K-mer length set to 25. A total of 48,033,980 and 53,409,010 raw reads were obtained from CK and IMD treatment groups, respectively. After removing adaptor sequences, low quality sequences and N-containing sequences, 46,760,944, and 51,668,492 clean reads were generated form the CK and IMD raw data, respectively. The assemblies produced 81,727 transcripts with a maximum sequence length of 19,224 bp and a N50 transcript length of 1,284 bp (Table 2). Furthermore, the GC content of the CK and IMD treatment groups were 31.47 and 30.76%, respectively. The quality of RNA samples and the expression file of genes were supplied as Table S2 and Datasheet 1.

**Table 2:** Assembly summary of the *A. gifuensis* transcriptome.

**Complete assembly**	**Samples**	
	**CK**	**IMD**
Total raw reads	48,033,980	53,409,010
Total clean reads	46,760,944	51,668,492
N50 transcript length	1,284 bp	
N90 transcript length	272 bp	
GC content	31.47%	30.76%

### Functional Gene Annotation and Classification

**Figure 2:**
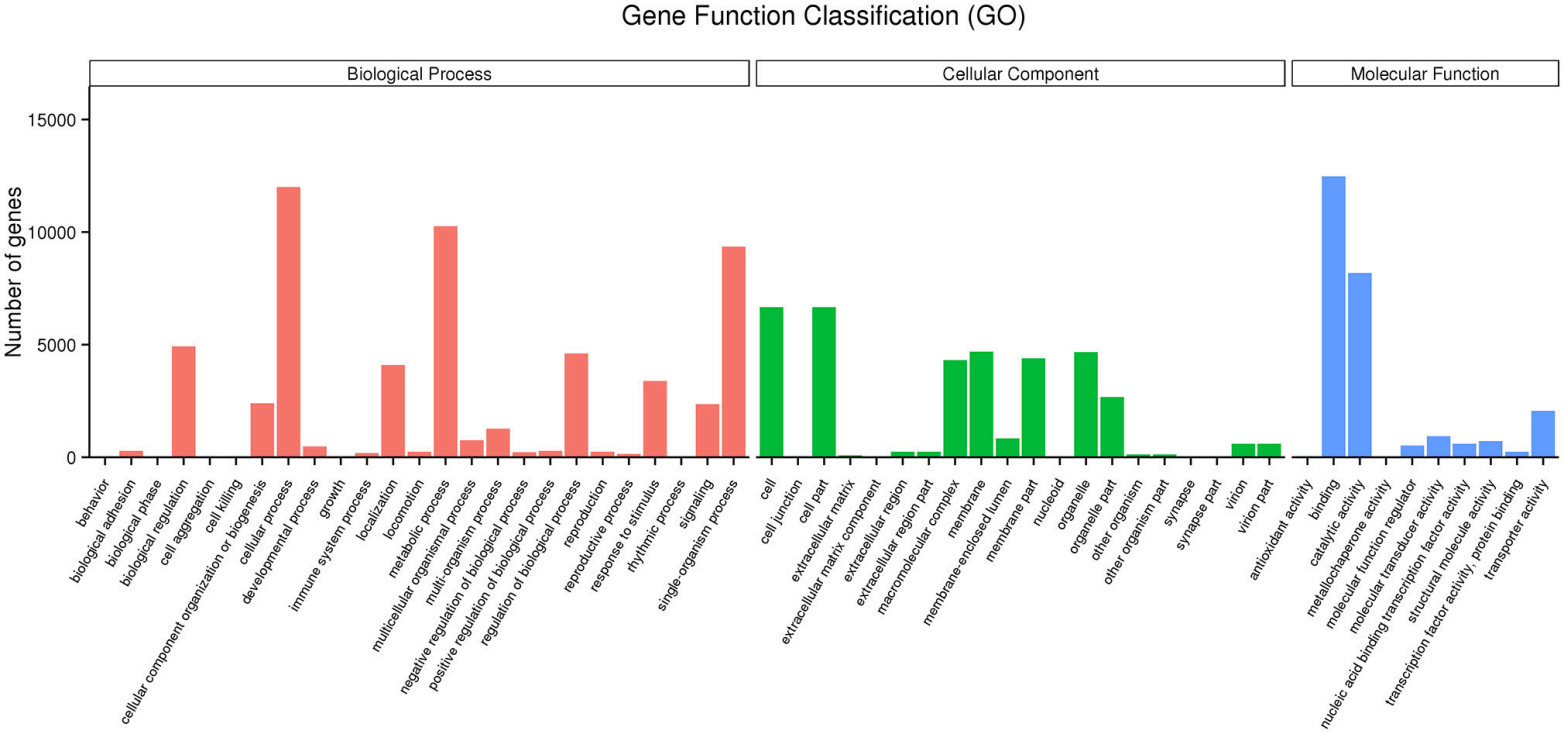
Functional annotation of *Aphidius gifuensis* transcripts based on gene ontology (GO) categorization.

**Figure 3:**
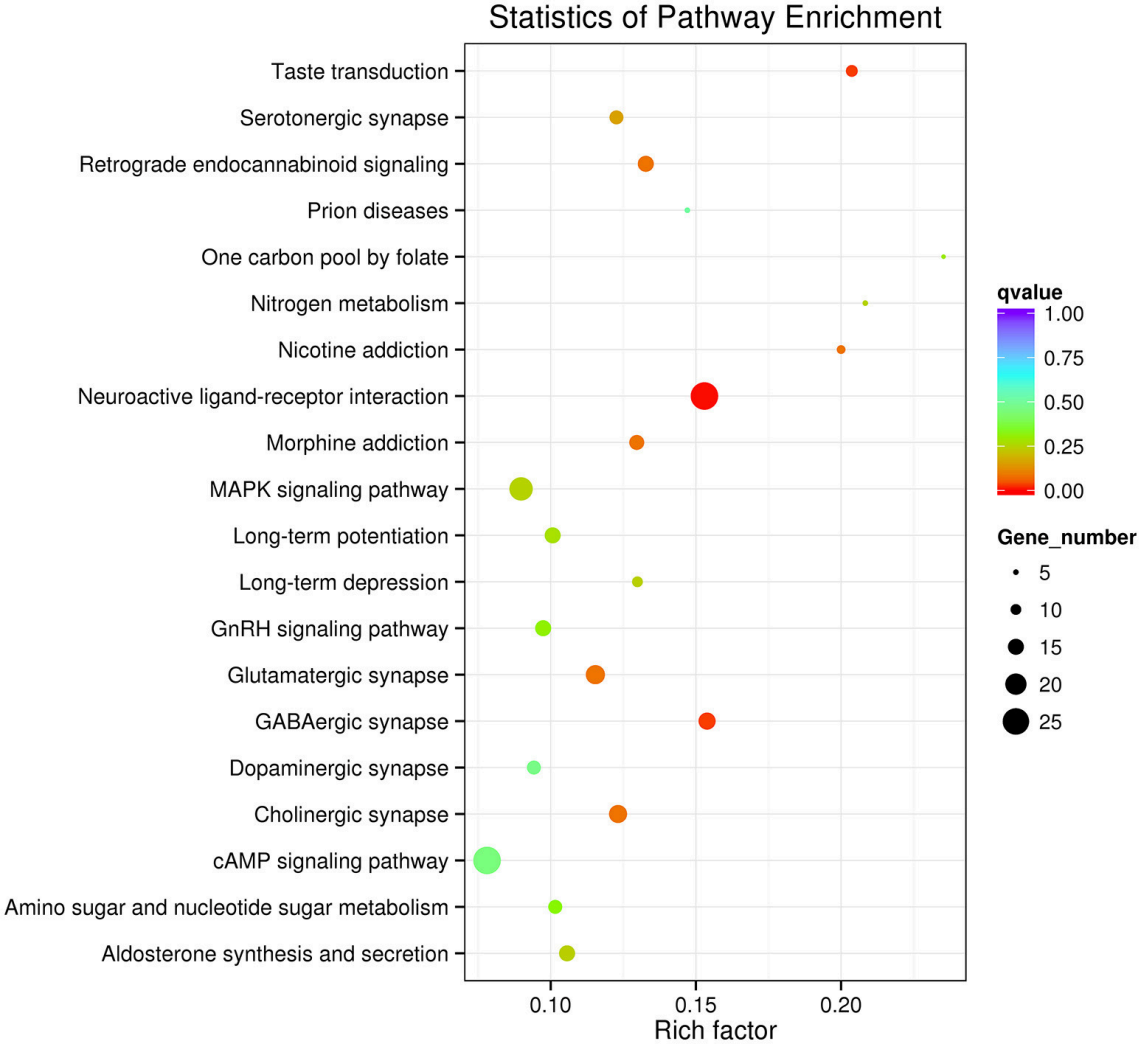
Top 20 enriched Kyoto Encyclopedia of Genes and Genomics (KEGG) pathways of *Aphidius gifuensis* after the exposure of sublethal does of IMD.

GO enrichment indicated that genes involved in the cellular process, metabolic process, single-organism process, biological regulation and the regulation of the biological process in the category of the biological process, cell, cell part, membrane and organelle in the category of cellular component, and binding and catalytic activity in the category of molecular function were dominant (Figure 2). The neuroactive ligand-receptor interaction, cAMP signaling pathway and MAPK signaling pathway were the major enrichment pathways in the IMD treatment group (Figure 3).

### Genes Involved in the Central Nervous and Olfactory Systems Are Differentially Expressed in Response to Sublethal Doses of IMD

The first gene groups we examined focused on the central nervous and olfactory systems, which are the target of the IMD and influence target insect behavior. For the target of the IMD, we identified 15 acetylcholine receptors: 11 neuronal acetylcholine receptors and four muscarinic acetylcholine receptors (Table 3). Among these target genes, *nAChR*α*4* and *nAChR*β*1* were significantly down-regulated in response to the IMD. Only *nAChR*α*7* and *nAChR*α*9* exhibited higher transcript abundances in the IMD treated *A. gifuensis* when compared to CK samples. Furthermore, no significant differences were detected in the rest of the *nAChRs* genes.

**Table 3:** The identified acetylcholine receptors in *A. gifuensis*.

**Gene name**	**Unigene reference**	Log_2_^*^	**Blast P hit**	***E*-value**	**Identify (%)**
**NEURONAL ACETYLCHOLINE RECEPTOR**					
*nAChRa1*	Cluster-9767.40203	0.11103	XP_015110344.1|PREDICTED: acetylcholine receptor subunit alpha-like 1 [*Diachasma alloeum*]	6e-90	69
*nAChRa2*	Cluster-9767.38760	0.13179	XP_014297158.1|PREDICTED: acetylcholine receptor subunit alpha-like 2 isoform X1 [*Microplitis demolitor*]	0	88
*nAChRa4*	Cluster-9767.22382	−1.3319	XP_008544424.1|PREDICTED: acetylcholine receptor subunit alpha-like isoform X1 [*Microplitis mediator*]	0	89
*nAChRa5*	Cluster-9767.30271	0.3851	XP_008555383.1|PREDICTED: neuronal acetylcholine receptor subunit alpha-5-like [*Microplitis demolitor*]	7e-175	56
*nAChRa6*	Cluster-9767.30497	0.34071	XP_008554100.2|PREDICTED: neuronal acetylcholine receptor subunit alpha-5-like [*Microplitis demolitor*]	3e-115	41
*nAChRa7*	Cluster-2049.3	1.5261	XP_015127141.1|PREDICTED: neuronal acetylcholine receptor subunit alpha-7 [*Diachasma alloeum*]	0	85
*nAChRa8*	Cluster-9767.29889	−0.0077548	XP_011307149.1|PREDICTED: neuronal acetylcholine receptor subunit alpha-7-like [*Fopius arisanus*]	0	92
*nAChRa9*	Cluster-9767.41854	1.6125	XP_023287837.1|neuronal acetylcholine receptor subunit alpha-5 isoform X1 [*Orussus abietinus*]	9e-90	40
*nAChRa10*	Cluster-9767.17337	0.55778	XP_008544424.1|PREDICTED: acetylcholine receptor subunit alpha-like isoform X1 [*Microplitis demolitor*]	0	92
*nAChRb1*	Cluster-9767.40511	−1.0589	EFN70707.1|Acetylcholine receptor subunit beta-like 1 [*Camponotus floridanus*]	4e-86	88
*nAChRb2*	Cluster-916.0	−0.016081	XP_015512110.1|PREDICTED: acetylcholine receptor subunit beta-like 2 [*Neodiprion lecontei*]	0	90
**MUSCARINIC ACETYLCHOLINE RECEPTOR**					
*mAChR1*	Cluster-9767.13216	0.37407	XP_008547263.1|PREDICTED: muscarinic acetylcholine receptor DM1 [*Microplitis demolitor*]	1e-76	54
*mAChR2*	Cluster-9767.25708	Inf	XP_011304151.1|PREDICTED: muscarinic acetylcholine receptor M2 isoform X2 [*Fopius arisanus*]	0	79
*mAChR3*	Cluster-9767.33389	0.011496	XP_014295111.1|PREDICTED: muscarinic acetylcholine receptor M3 [*Microplitis demolitor*]	8e-23	57
*mAChR4*	Cluster-2018.0	Inf	XP_011304149.1|PREDICTED: probable muscarinic acetylcholine receptor gar-1 isoform X1 [*Fopius arisanus*]	1e-40	78

* *Log_2_, Log_2_IMD/CK. Inf means this gene only expressed in IMD treated A. gifuensis*.

**Figure 4:**
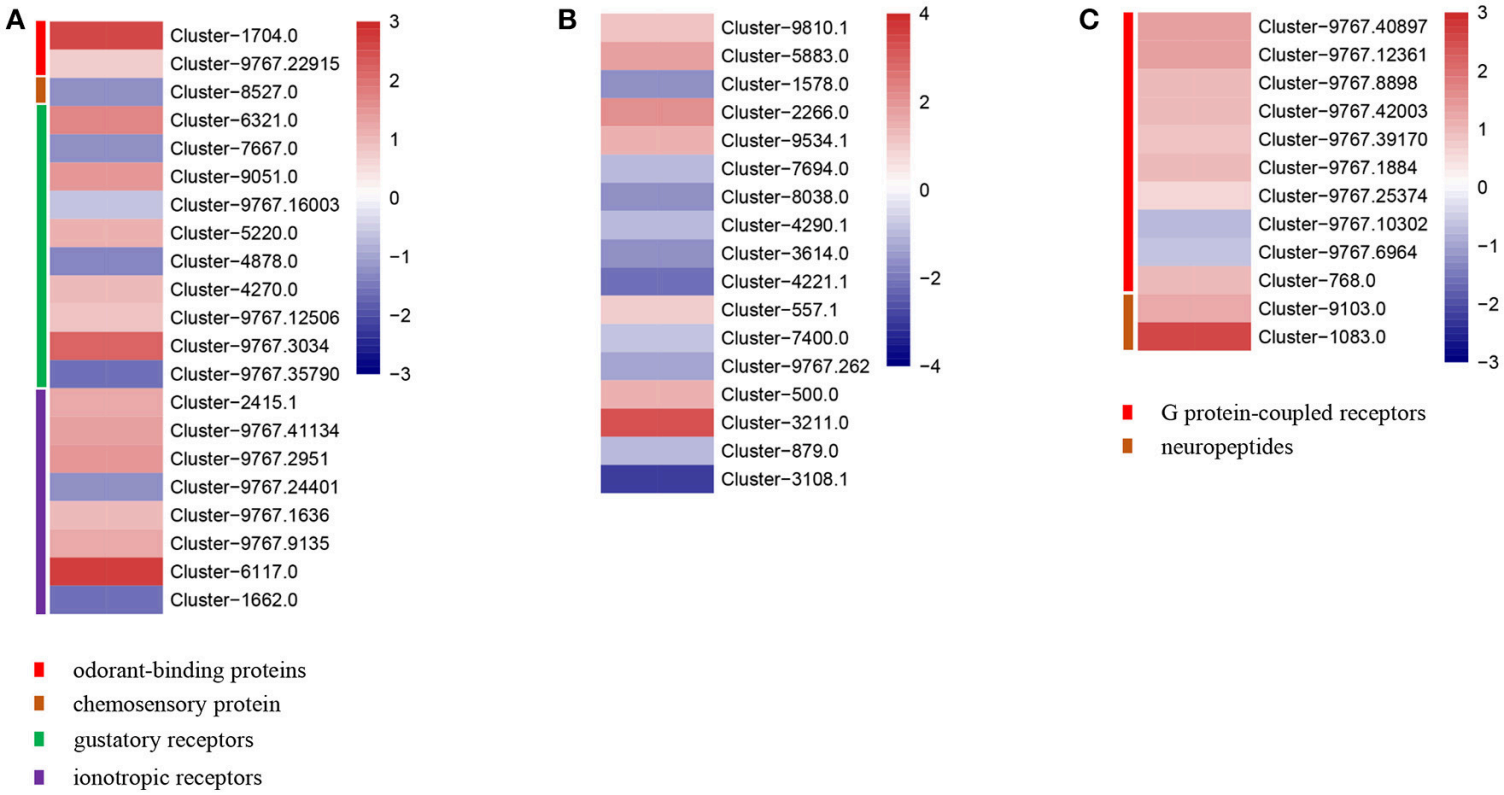
Heatmaps of expressions for genes with neuron and odorant related functions. **(A)** Genes identified as odorant-binding proteins, chemosesory proteins, gustatory receptors, and ionotropic receptors. **(B)** Genes identified as odorant receptors. **(C)** Genes involved in neuron functions.

Apart from the potential target genes, we also analyzed the impact of IMD exposure on olfactory systems to explain the impaired orientation behavior we observed in *A. gifuensis* treated with IMD. We found that a very low proportion of olfactory related genes exhibited significant differences between the treatments and control samples (Figure 4). The decrease in the mean FPKM values for the odorant co-receptor (Cluster-8038.0), odorant receptors (Cluster-1578.0, Cluster-4221.1, and Cluster-3108.1), chemosensory protein (Cluster-8527.0), gustatory receptors (Cluster-7667.0 and Cluster-4878.0), and ionotropic receptors (Cluster-9767.24401 and Cluster-1662.0) was particularly striking. On the contrary, exposure to IMD significantly up-regulated the expression of the odorant-binding protein (Cluster-1704.0), odorant receptor (Cluster-3211.0), gustatory receptor (Cluster-9767.3034), and the ionotropic receptor (Cluster-6117.0), which were effected the most by IMD treatment in their gene group.

Furthermore, exposure to IMD also influenced the expression of genes involved in the central neurons. The dopamine receptor 1 (Cluster-9767.1884), tryptophan 5-hydroxylase (Cluster-1083.0), neuropeptide FF receptor (Cluster-9767.40897) were significantly higher in IMD treatments compared to CK treatments.

### Effects of Sublethal Doses of IMD on Detoxification Progress, Antioxidant System, and Biomolecule Damage Genes in *A. gifuensis*

**Figure 5:**
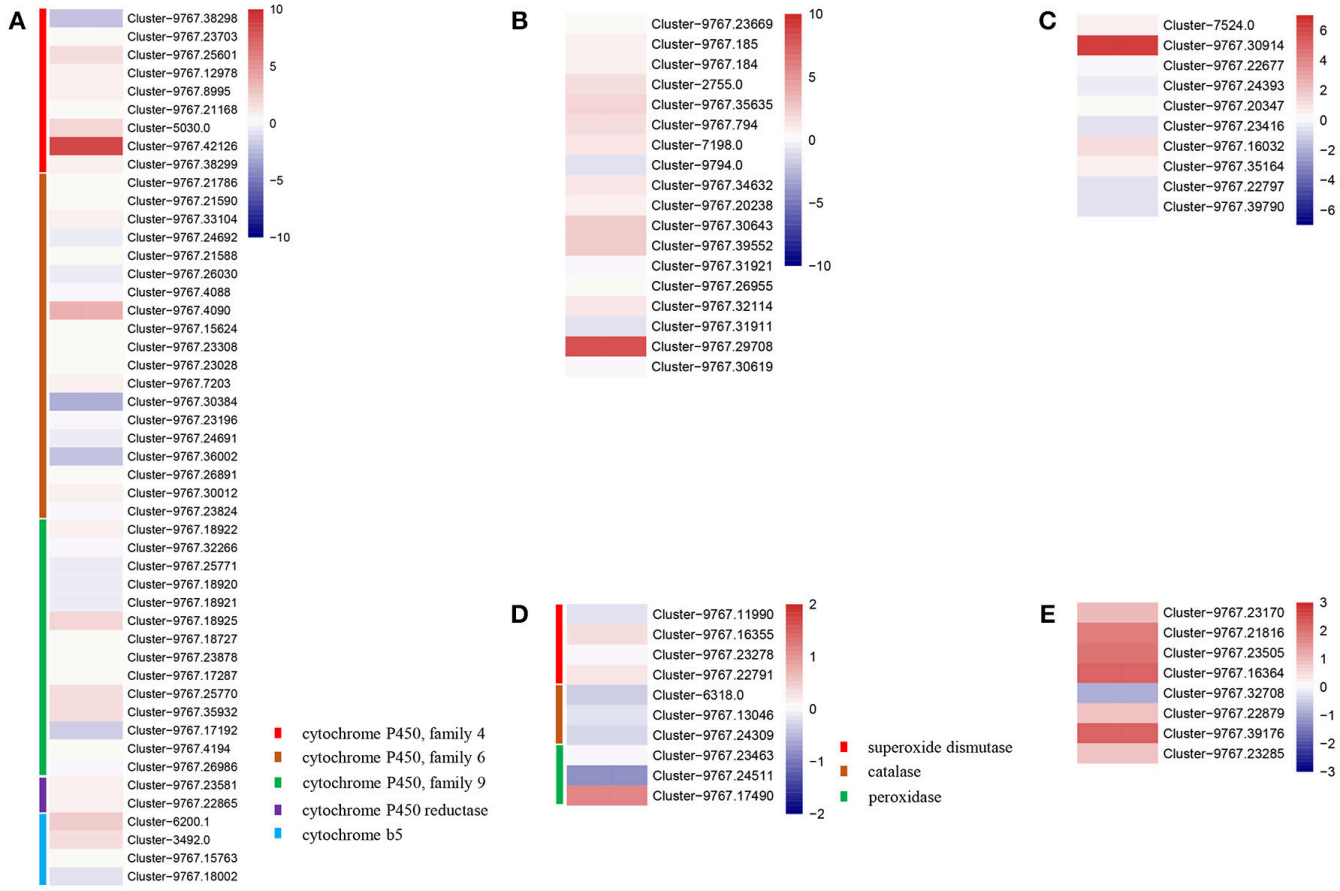
Heatmaps of expressions for genes with detoxification and stress response. **(A)** Genes involved in cytochrome P450. **(B)** Genes identified as carboxylesterase. **(C)** Genes identified as glutathione S-transferees. **(D)** Genes involved in antioxidant enzymes. **(E)** Gene identified as heat-shock proteins.

We found that defense genes, such as cytochrome P450 (CYP4c1: Cluster-5030.0 and Cluster-9767.42126; CYP6a2: Cluster-9767.4090; CYP9p3: Cluster-9767.18925), cyt b5 (Cluster-6200.1), peroxidase (POD: Cluster-9767.17490), carboxylesterase (CarE, Cluster-9767.29708), glutathione S-transferaes (GSTs, Cluster-9767.30914), and heat shock proteins (HSPs, Cluster-9767.16364, and Cluster-9767.39176), were highly expressed in the IMD treated *A. gifuensis* (Figure 5), while three P450s (Cluster-9767.38298, Cluster-9767.30384, and Cluster-9767.36002), POD (Cluster-9767.24511), and HSP (Cluster-9767.32708) exhibited lower transcript abundances in the IMD treated *A. gifuensis* than in the CK group (Figure 5).

### Sublethal Doses of IMD Altered Expression of Genes Involved in Metabolic Signaling

**Figure 6:**
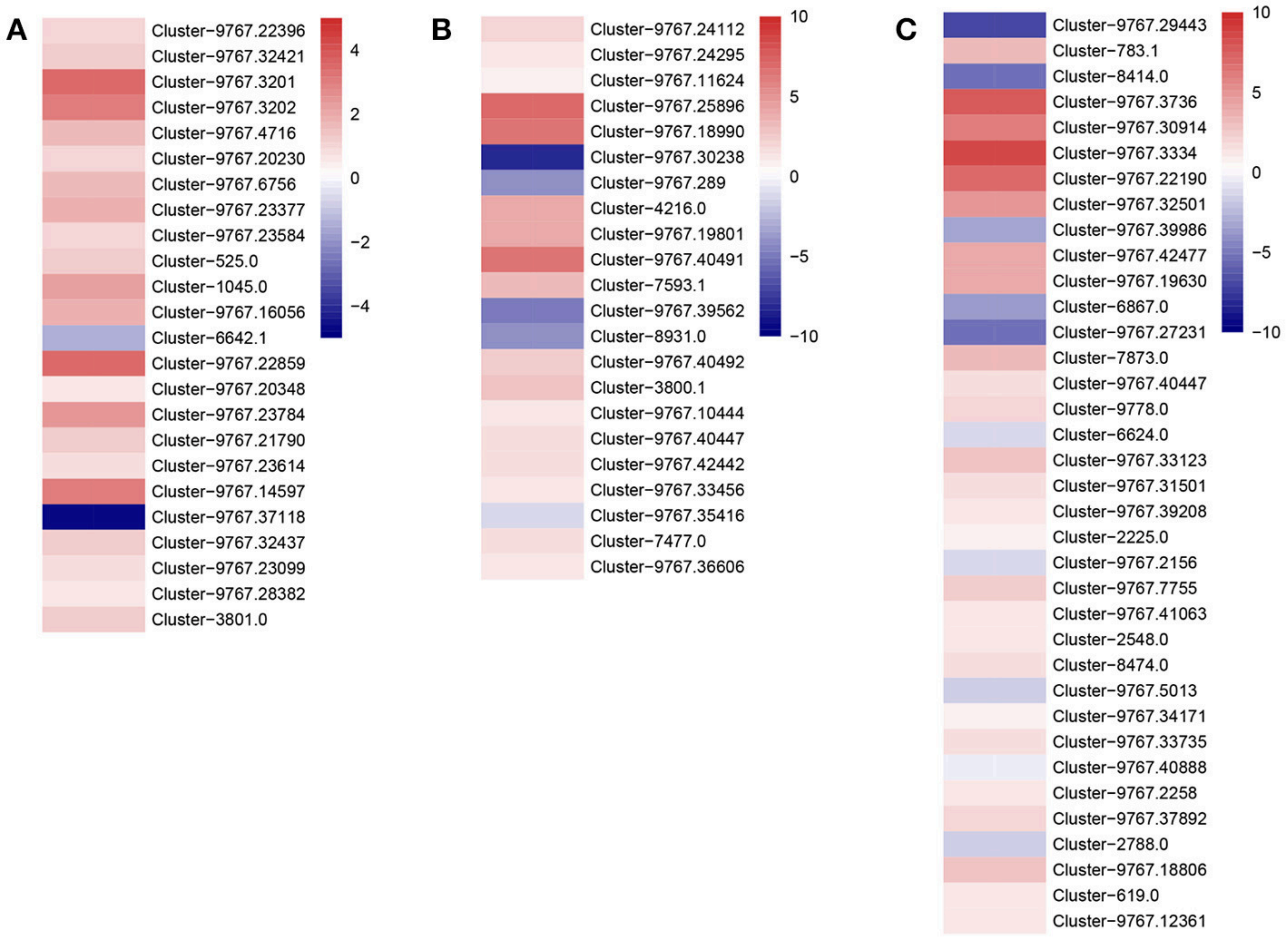
Heatmaps of expressions for genes with energy metabolic signaling. **(A)** Genes with functions in fatty acid metabolism. **(B)** Genes with functions in sugar metabolism. **(C)** Genes with functions in amino acid metabolism.

To investigate the impact of IMD on energy metabolism, we analyzed the expression of genes involved in fatty acid, sugar, and amino acid metabolism (Figure 6). We found that almost all the genes involved in fatty acid metabolism were expressed at a higher level in the IMD treated *A. gifuensis* (Figure 6A), while only Cluster-9767.37118 and Cluster-6642.1 were expressed at a lower level of the IMD treatment. Consistent with fatty acid metabolism, the majority of genes that regulate sugar and amino acid metabolism, also exhibited higher mean FPKM values in the IMD treated *A. gifuensis*, whereas the expression of Cluster-9767.30238, Cluster-9767.39562, Cluster-8931.0, and Cluster-9767.35416 in sugar metabolism and Cluster-2788.0, Cluster-6624.0, Cluster-6867.0, Cluster-9767.27231, Cluster-9767.29443, Cluster-9767.39986, and Cluster-9767.5013 in amino acid metabolism, were down-regulated in response to the IMD treatment (Figures 6B,C).

### Validation of Transcriptome Data by qPCR

**Figure 7:**
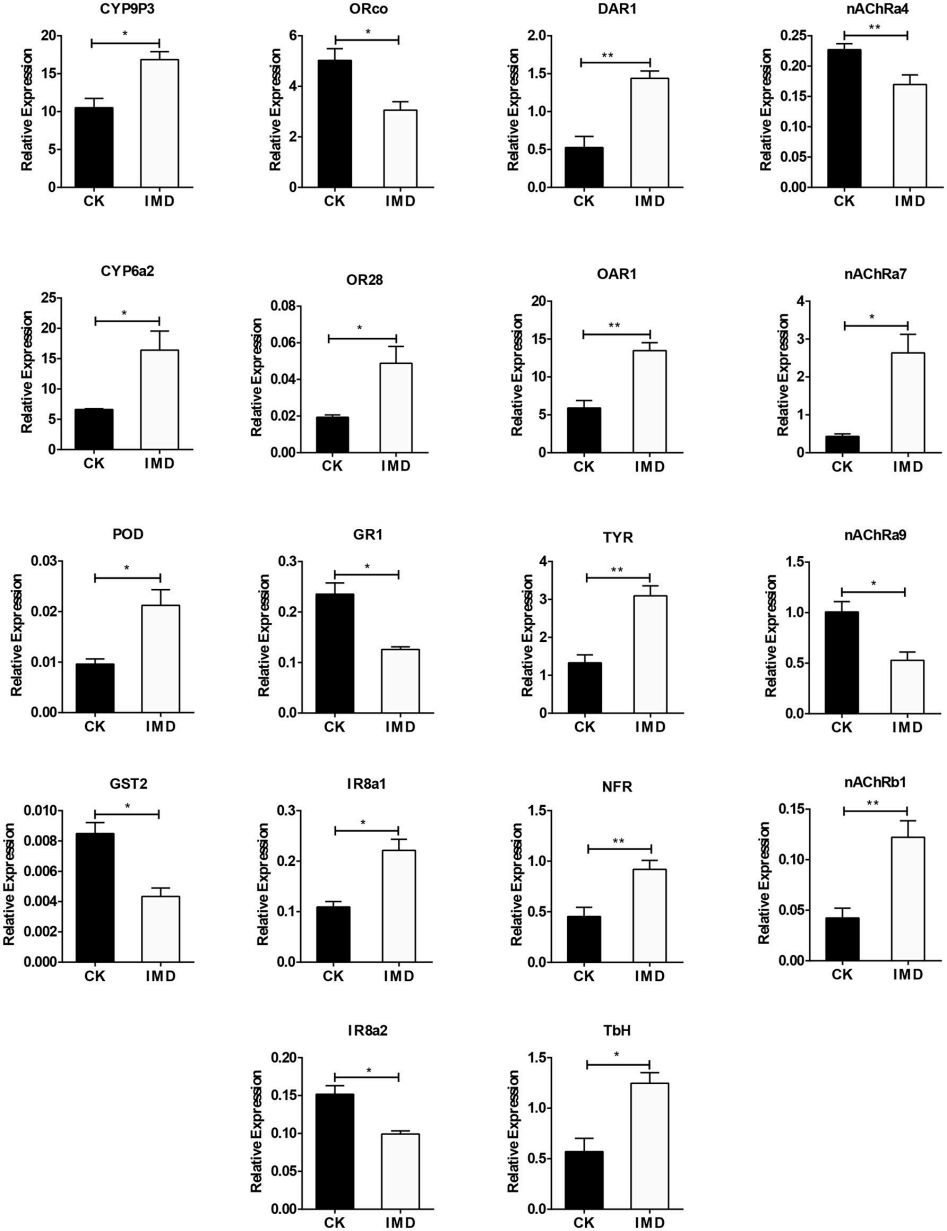
Validation of expression of selected genes using qPCR. DAR1, dopamine receptor 1; SNF, short neuropeptide F; NFR, neuropeptide FF receptor 2; TYR, putative tyramine receptor 2; TbH, tyramine beta-hydroxylase. Asterisk over the bars designate a significant difference based on student's *t*-test: ^*^P < 0.05, ^**^P < 0.01, ^***^P < 0.001, and the error bars is ± SE bars. *N* = 3.

To confirm the transcriptome data, we conducted the RT-qPCR of several genes identified in the transcriptome that were IMD-sensitive. Exposure to IMD significantly increased the expression of *CYP6a2, CYP9P3, POD, OR28, IR8a1, nAChRa7, nAChRa9, TbH, OAR1, NFR, TYR*, and *DAR1*, whereas the expression of *GST2, nAChRa4, nAChRb1, ORco, IR8a2*, and *GR1* decreased (Figure 7). Furthermore, exposure to IMD did not influence the expression of *GST5, SOD1*, and *SOD2* (Figure S1).

### Activities of CarEs, POD, and GSTs in *A. gifuensis* After IMD Exposure

**Figure 8:**
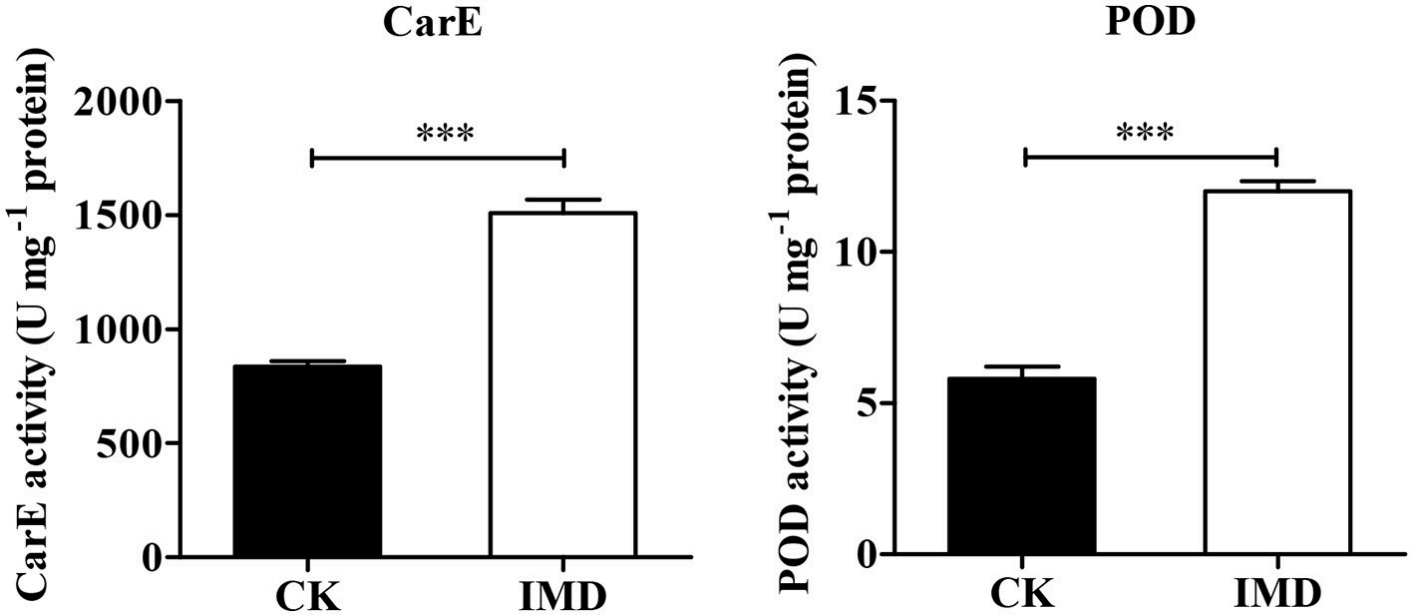
Activities of CarE and POD. Asterisk over the bars designate a significant difference based on student's *t*-test: ^*^P < 0.05, ^**^P < 0.01, ^***^P < 0.001, and the error bars is ± SE bars. *N* = 3.

Exposure to IMD significantly induced the activities of POD and CarEs, while it had no significant influence on SOD, CAT and GST activity (POD: *t* = −11.648, *df* = 4, *P* < 0.001; CarE: *t* = −10.552, *df* = 4, *P* = 0.003; SOD: *t* = 0.843, *df* = 4, *P* = 0.4467; CAT: *t* = 0.6523, *df* = 4, *P* = 0.2298; GST: *t* = 1.886, *df* = 4, *P* = 0.1323; Figure 8 and Figure S2).

## Discussion

IPM program improvements requires an understanding of how pesticides influence natural enemies of the pests that are being targeted. Therefore, the effects of sublethal doses of pesticides are important for improving IPM programs. In this work, we found that oral ingestion of sublethal doses of IMD, adversely affected parasitoid performance, including the survival rate, parasitic capacity, and longevity of female adults, which was consistent with the performance of the *Aphidius colemani, Microplitis mediator, O. insidiosus, C. flavipes*, and *Trichogramma* species exposed to pesticides (D'Avila et al., 2018; Fontes et al., 2018). In the *M. mediator*, exposure to flonicamid, pymetrozine, spinosad, and thiacloprid reduced its parasitization activity, percentage of parasitism and female longevity. In addition, IMD impaired the longevity and parasitic capacity of *Trichogramma* species including *Trichogramma achaeae, T. chilonis, T. platneri*, and *T. pretiosum* (Khan and Ruberson, 2017; Fontes et al., 2018). The exposure to pesticides also adversely affected the biocontrol efficiency of pest predators (Moscardini et al., 2013; Nawaz et al., 2017). For example, IMD significantly repressed egg hatching, nymph survival and adult fecundity of the predatory bug, *Orius albidipennis* (Sabahi et al., 2010; Moscardini et al., 2013). Similarly, sublethal doses of diazinon, fenitrothion, and chlorpyrifos exhibited adverse effects on the biological performance of *Andrallus spinidens*, which is a predator of rice lepidopterous larvae (Gholamzadeh-Chitgar et al., 2015). Similar to chemical pesticides, other biological agents like entomopathogenic fungi and bacteria also adversely affect the biological performance of parasitoids and predators (Potrich et al., 2017). In addition, a high occurrence of wing deformities was observed when mummies of *A. gifuensis* were exposed to IMD (44.44%), acetamiprid (67.25%), and thiamethoxam (33.33%) (Sun et al., 2014). All of these results indicated that pesticide exposure adversely influenced the performance of natural enemies, which also means that the effectiveness of natural enemies can be reduced by the application of pesticide.

In addition to biological performance, we also investigated the side effects of IMD on the orientation behaviors of *A. gifuensis* after IMD treatment. We found that exposure of IMD significantly reduced the sensitivity of *A. gifuensis* to the volatiles produced by aphid infested plants. Consistent with this results, consuming IMD or aldicarb contaminated floral nectar, also reduced the response of *Microplitis croceipes* to the odors from its host *Helicoverpa zea* infested cotton (Stapel et al., 2000). In *Anagrus nilaparvatae*, survivors of IMD exposure had no response to volatiles from *Nilaparvata lugens*-infested rice (Liu et al., 2010). In addition, exposure to pyrethroids impaired the host-searching and oviposition behavior of the aphid parasitoids *Aphidius ervi* and *Aphidius colemani*, and *Trissolcus basalis*, which is an egg parasitic wasp of the southern green stinkbug*, Nezara viridula* (Ahmad and Hodgson, 1998; Salerno et al., 2002; Desneux et al., 2004a). Furthermore, Wang D. et al. (2017) found that exposure to beta-cypermethrin significantly decreased pheromone perception in male *Trichogramma chilonis*. All of these results indicate that IMD exposure impairs or reduces the sensitivity of the *A. gifuensis* olfactory system, thereby disrupting host searching and parasitizing.

To explore the potential mechanism of the negative effects of IMD on *A. gifuensis*, transcriptome technology was used to comprehensively analyze the gene expression of *A. gifuensis* in response to sublethal doses of IMD exposure. Our transcriptomic analysis pointed to a profound regulation of genes principally related to the olfactory and neuronal systems. The most down-regulated genes were the odorant co-receptor (Cluster-8038.0), which is the most important odorant receptor in the detection of odorants; gustatory receptor 1 (Cluster-7667.0), a sugar receptor that is associated with host aphid discrimination; and the neuropeptides capa receptor (Cluster-9767.10302), a G protein-coupled receptor for the capa peptides and an important signaling molecule in the regulation of a wide range of physiological processes (Kang et al., 2017b; Schoofs et al., 2017). These results are generally consistent with recent studies of the interaction of neonicotinoid with OBPs and CSPs. For example, CSP3 and OBP21 were downregulated in honey bees exposed to thiamethoxam (Shi et al., 2017). Furthermore, a sublethal dose of IMD inhibited the binding affinity of OBP2 and CSP1 to a floral volatile β-ionone in *Apis cerana* and *GOBP2* to a tea volatile *E*-2-hexenal in *Agrotis ipsilon* (Li et al., 2015, 2017a,b). Interestingly, in addition to these down-regulated genes, a considerable number of genes were up-regulated, such as the odorant-binding protein (Cluster-1704.0), gustatory receptor (Cluster-9767.3034), ionotropic receptor (Cluster-6117.0), and odorant receptor (Cluster-3211.0). Similarly, a single brief exposure to pesticides dramatically increased CSP expression in *Bombyx mori* (abamectin) and *Bemisia tabaci* (thiamethoxam) (Xuan et al., 2015; Liu G. et al., 2016). All of these results indicate that the impairment of olfactory systems from sublethal doses of some pesticides could be involved the disordered orientation behavior.

As a neurotoxin and agonist of nAChRs, IMD had high binding affinity for nAChRs, thereby disrupting neurotransmission (Ffrench-Constant et al., 2016). IMD was thought to impede information receiving and processing in *N. vitripennis*, which led to the disruption of sexual communication and foraging behavior (Cook et al., 2016; Tappert et al., 2017). Furthermore, in *Solenopsis invicta*, when treated with 0.25 μg/ml IMD, there was a significant reduction in food consumption, digging and foraging behavior, while the neurotoxins flubendiamide and indoxacarb increased the walking time of *Copidosoma truncatellum* (Wang L. et al., 2015; Ramos et al., 2018). In the current work, we found that *nAChRa4* and *nAChRb1* were down-regulated, which was consistent with the expression of nAChRs in *Rhopalosiphum padi* (Wang K. et al., 2017). Similar to the findings of Desneux et al. (2004b) in *A. ervi*, we also found that some of the IMD exposed females bended their abdomen forward as they were attacking aphid, while no aphids were present. All of these results revealed that sublethal doses of IMD not only impaired the olfactory system of *A. gifuensis*, but also disrupted the neurotransmission that influences their behavior. Furthermore, these results also indicate that the development of specific and environmentally safe pesticides, that present little or no harm to natural enemies of pest insects, are needed.

In addition to the effect IMD had on the olfactory and neuron systems, we also investigated the impact of IMD on the detoxification systems in *A. gifuensis*. Cytochrome P450 monooxygenases (P450s), carboxyl esterases (CarEs), and glutathione S-transferees (GSTs) are three major multiline enzyme families that are responsible for xenobiotic metabolism in most insect species (Li et al., 2007; Hsu et al., 2012; Chaimanee et al., 2016; Gong and Diao, 2017; Magesh et al., 2017; Traverso et al., 2017).

P450s are a group of important stress response-related genes that play significant roles in several physiological processes, including hormone metabolism, the adaptation to natural and synthetic toxins, and insecticide detoxification. As we know, overexpression of the gene coding of the P450 clades (CYP4, CYP6, and CYP9), contributes considerably to insecticide-resistance (Li et al., 2007; Bass et al., 2014). For example, in *B. tabaci* and *M. persicae*, over-expression of the cytochrome P450 genes *CYP6CM1* and *CYP6CY3*, contribute to neonicotinoid insecticide resistance, as these enzymes can catalyze a more rapid conversion of imidacloprid to its less active form, 5-hydroxy-imidacloprid (Karunker et al., 2008; Puinean et al., 2010). Furthermore, *CYP6AY1* and *CYP6ER1* were highly overexpressed in the IMD resistant strain of *N. lugens* compared to the susceptible strain (Yang Y. X. et al., 2017). In *A. mellifera*, coumaphos and IMD treatment significantly decreased the expression of *CYP306A1, CYP4G11*, and *CYP6AS14*, whereas pyrethroid bifenthrin induced the expression of *CYP9Q1* and *CYP9Q2* but repressed the expression of *CYP9Q3* (Mao et al., 2011; Chaimanee et al., 2016). *In-vitro, CYP9Q1, CYP9Q2*, and *CYP9Q3* detoxify coumaphos independently and tau-fluvalinate with the cooperation of CarEs (Mao et al., 2011). Additionaly, *CYP9Q1* and *CYP9Q3* also contributed to the metabolism of quercetin (Mao et al., 2011). In this work, the most up-regulated genes were *CYP4c1* and *CYP6a2* (Cluster-9767.42126), which are associated with the IMD resistance in *N. lugens*; and *cyt b5* (Cluster-6200.1), which is the electron transfer partners of P450 proteins and which modify the catalytic activity of P450 proteins (Paine et al., 2005; Ding et al., 2013).

CarEs are involved in the metabolic detoxification of dietary and environmental xenobiotics in insects (Xie et al., 2017; Wu et al., 2018). A higher expression or activity of CarEs have been reported in the insecticide resistance strains of many insect species such as *M. persicae, R. padi, Aphis gossypii, Pediculus humanus capitis, P. xylostella*, and *Bactrocera dorsalis* (Hsu et al., 2012; Gong et al., 2013, 2014; Kwon et al., 2014; Wang L. et al., 2015; Wang L. L. et al., 2015; Xie et al., 2017). In *A. mellifera*, the induction of CarE activity by IMD, acetamiprid, pymetrozine, and pyridalyl was observed, while malathion and permethrin significantly inhibited CarE activity (Yu et al., 1984; Suh and Shim, 1988; Badawy et al., 2014; Li Z. et al., 2017). Furthermore, in *P. xylostella*, CarEs activity was positively correlated with resistance to spinosyn, beta-cypermethrin, chlorpyrifos, and abamectin (Gong et al., 2013). Moreover, RNA interference-mediated gene silencing (RNAi) tests revealed that the knock-down of *CarE* genes led to a decreased tolerance to some pesticides (Wang L. L. et al., 2015). In *B. dorsalis*, the knock-down of *CarE4* and *CarE6* significantly decreased the resistance to malathion, and the detoxification of malathion was observed when *CarE4* and *CarE6* were heterologously expressed (Wang L. L. et al., 2015). Furthermore, in *Lygus lineolaris*, IMD exposure significantly increased the expression of 13 esterase genes (Zhu and Luttrell, 2015). In this work, we found that the expression and enzyme activity of CarEs in IMD treated *A. gifuensis* were significantly higher than that in CK treatment, especially carboxylesterase (Cluster-9767.29708).

GSTs are part of another important detoxification enzyme family. GST activity in larvae, pupae, and nurse bees, but not in foragers, was induced by pyrethroid flumethrin (Nielsen et al., 2000). In the eastern honey bee *Apis cerana cerana*, the sigma-class *AccGSTS1* was up-regulated by phoxim, cyhalothrin and acaricide and the theta-class GST gene *GSTT1* and omega-class GST gene *GSTO2* was induced by cyhalothrin, phoxim, pyridaben, and paraquat, indicating that they might be involved in the stress response to pesticides (Yan et al., 2013; Zhang et al., 2013; Liu S. et al., 2016). Furthermore, formetanate increased the activity of GST, whereas IMD and dimethoate had no influence on GST activity in *A. mellifera* (Li Z. et al., 2017; Staron et al., 2017). However, in this work, only one GST (Cluster-9767.30914) was found to be highly expressed in the IMD treated *A. gifuensis* compared to the CK treatment, while the rest of the GSTs did not show any response to sublethal doses of IMD treatment. Similarly, in *Lygus lineolaris*, only four of the 19 GSTs were significantly down-regulated after IMD exposure, while the rest of these genes did not show any detectable difference in expression (Zhu and Luttrell, 2015). All of these results indicate that GST might not be responsible for IMD resistance in *A. gifuensis*.

In addition to these three major detoxification pathways, other interrelated pathways might also contribute to the response of xenobiotics, such as superoxide dismutase (SOD), catalase, POD, and HSPs (Chaimanee et al., 2016). In honeybee queens, exposure to IMD and coumaphos significantly depressed the expression of SOD and thioredoxin peroxidase (Chaimanee et al., 2016). Conversely, the expression of catalase, SOD and thioredoxin peroxidase was significantly increased in worker bees (Chaimanee et al., 2016). In this work, POD (Cluster-9767.17490) and HSPs (Cluster-9767.16364 and Cluster-9767.39176) were up-regulated in IMD treated *A. gifuensis*, which is consistent with the HSP expression profiles in beta-cypermethrin treated *R. padi* (Li Y. T. et al., 2017). Further, exposure to IMD significantly decreased the expression of GSTs. Together, these results indicate that detoxification and stress response systems are critical for protecting *A. gifuensis* from IMD damage.

To support or drive detoxification processes, the increased energy production through the up-regulation of enzymes involved in ATP synthesis, sugar metabolism, fatty acid metabolism, glycolysis, and the tricarboxylic acid (TCA) cycle were investigated. Our transcriptome data revealed that IMD treatment altered the expression of genes in energy-producing metabolic pathways such as fatty acid metabolism and sugar metabolism. Consistent with this finding, exposure to neonicotinoid also led to increased energy usage in honey bees (du Rand et al., 2017). Furthermore, exposure to a sublethal dose of beta-cypermethrin, led to an increase in respiratory quotient and respiratory rates in *Harmonia axyridis*, which is often coupled with the status of energy metabolism (Xiao et al., 2017). All of these results suggest that insects increase their energetic cost when undergoing detoxification after pesticide exposure. The increase in their energetic cost might result in the decrease of longevity and parasitism.

With the wide use of pesticides in agriculture and horticulture, understanding how pesticides impair, and influence biological efficiency of natural enemy insect species and how natural enemies adjust their detoxification mechanisms to metabolize pesticides is very important. In this work, we found that exposure to sublethal doses of IMD significantly affected the biological performance of *A. gifuensis*, potentially through changes in the expression of genes involved in the nervous, olfactory, detoxification systems and energy metabolism. Our results indicated that pesticides may block some physiological or biochemical processes that lead to the disruption of the survival, growth, development, reproduction, and behavior of the natural enemies of insect pests. Based on these results, we not only elucidated the sublethal effects of pesticides on the natural enemies, but also contributed to a better understanding of how residual pesticides influence the biological performance of natural enemies and how natural enemies respond to environmental xenobiotics. Our results provide an insight on how to improve experimental approaches, to investigate IPM.

## Author Contributions

Z-WK and T-XL designed the study. Z-WK, R-PP, and F-HL performed research. Z-WK, F-HL, and H-GT analyzed data. Z-WK wrote the manuscript. H-GT and T-XL edited the manuscript. Z-WK revised the manuscript.

### Conflict of Interest Statement

The authors declare that the research was conducted in the absence of any commercial or financial relationships that could be construed as a potential conflict of interest.

## Supplementary Material

Figure S1 qPCR results of SNF, GST5, SOD1 and SOD2. ns over the bars mean no significant difference, and the error bars is ± SE bars. *N* = 3.

Figure S2 Activities of SOD, CAT, and GST. ns over the bars mean no significant difference, and the error bars is ± SE bars. *N* = 3.

Table S1 Primers used for target genes and reference genes in qPCR.

Table S2 RNA quality of transcriptomic samples.

Datasheet 1 Gene expression differences between IMD and CK.
