# Supplementary figures and images for: Effect of Sublethal Doses of Imidacloprid on the Biological Performance of Aphid Endoparasitoid Aphidius gifuensis (Hymenoptera: Aphidiidae) and Influence on Its Related Gene Expression
Source: Front Physiol. 2018 Dec 11;9:1729. doi: 10.3389/fphys.2018.01729 (PMC6297876; doi:10.3389/fphys.2018.01729)

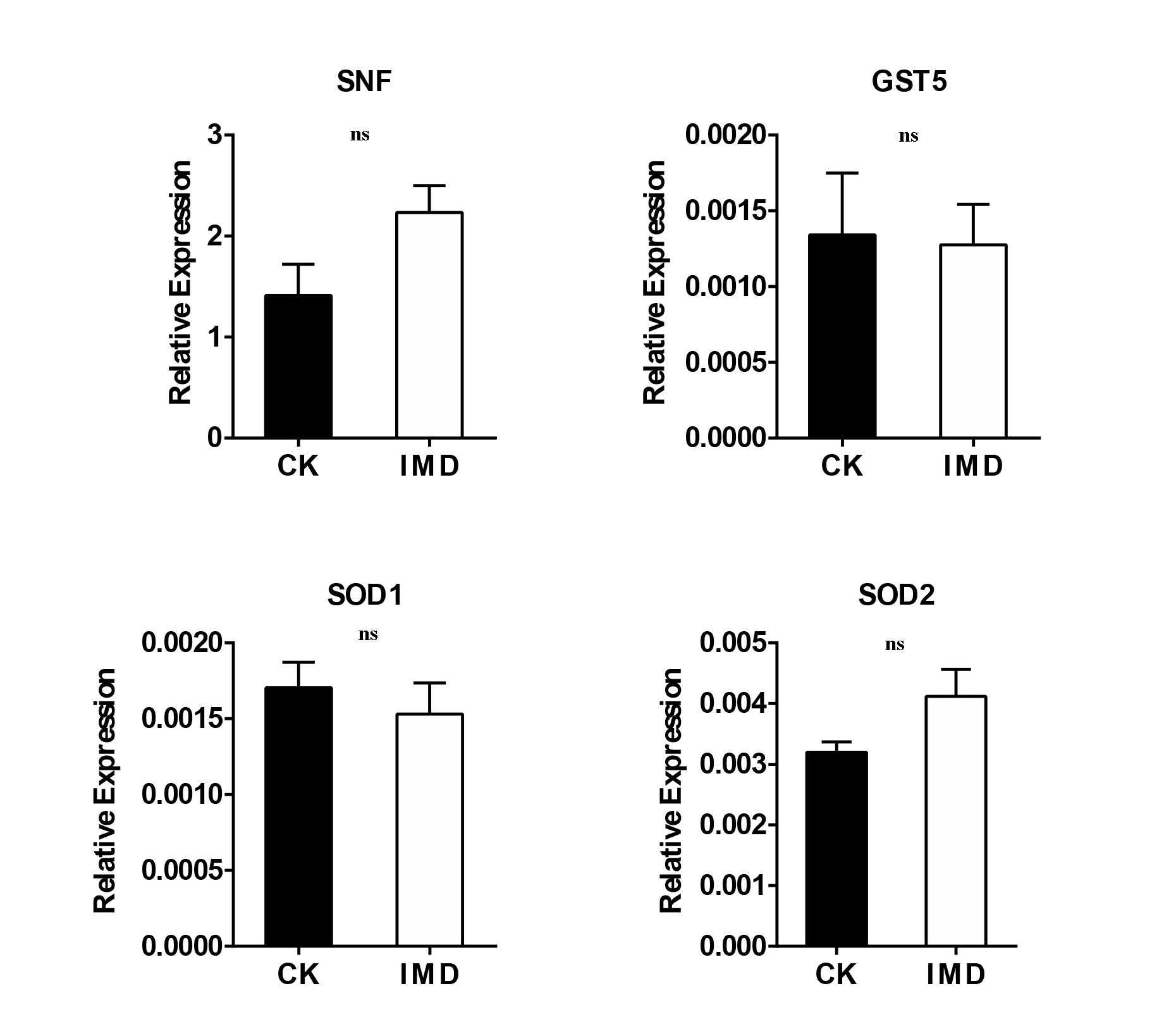

Supplement: Figure S1 — qPCR results of SNF, GST5, SOD1 and SOD2. ns over the bars mean no significant difference, and the error bars is ± SE bars. N = 3. [file Image_1.tif]

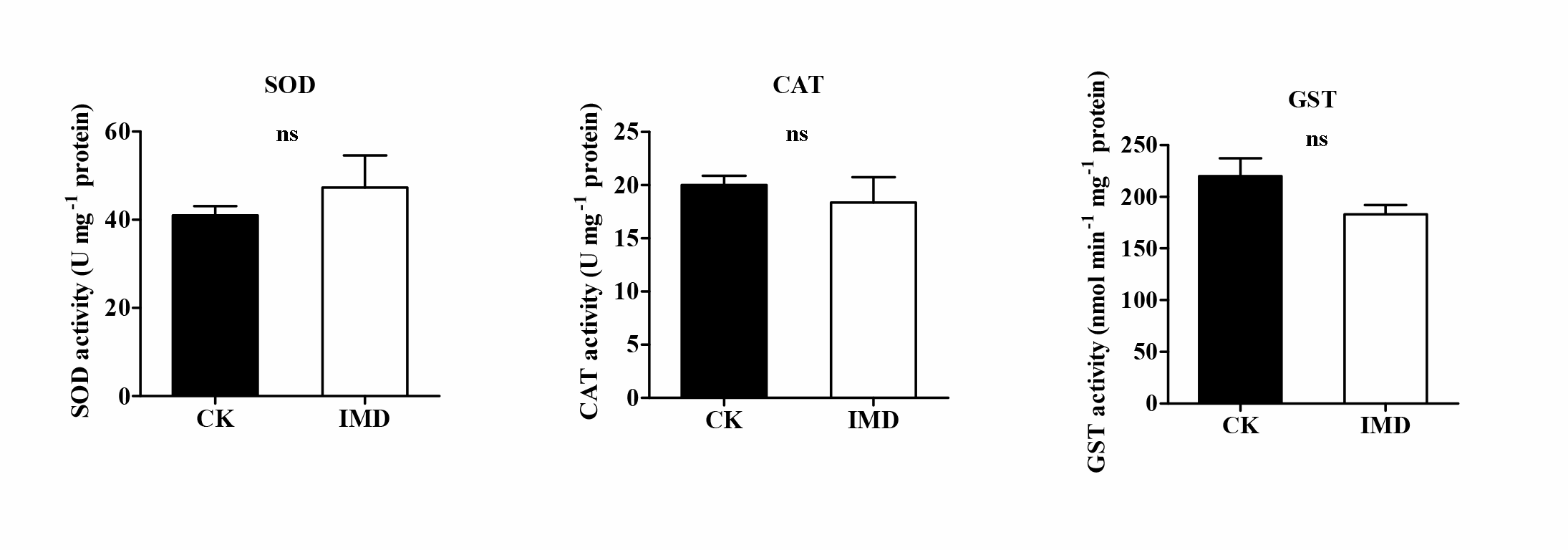

Supplement: Figure S2 — Activities of SOD, CAT, and GST. ns over the bars mean no significant difference, and the error bars is ± SE bars. N = 3. [file Image_2.tif]
