# Supplementary material for: Effect of Sublethal Doses of Imidacloprid on the Biological Performance of Aphid Endoparasitoid Aphidius gifuensis (Hymenoptera: Aphidiidae) and Influence on Its Related Gene Expression
Source: Front Physiol. 2018 Dec 11;9:1729. doi: 10.3389/fphys.2018.01729 (PMC6297876; doi:10.3389/fphys.2018.01729)
Supplement: Table S1 — Primers used for target genes and reference genes in qPCR. [file Table_1.DOCX]

**Table S1. The primers used in this study.**

| Name | Primers | Size |
| --- | --- | --- |
| *CYP9p3* | F: GGATTGGGTCCACGTAAATGTATCG | 177 |
|  | R: CCACCAGCTGTAATGTAAAGTGGT |  |
| *CYP6a2* | F: ATGGAGCTGGGTGTTGTTGAATTG | 116 |
|  | R: GGTTTTGGTCCAGGAATACCCAGTT |  |
| *DAR1* | F: CCTTGTTATCTGAGCTGTTTGTGGG | 234 |
|  | R: CACAAAAACGTGGACCAAACATCC |  |
| *SNF* | F: ATTGGTTTTGCAGTGGCTAGAC | 197 |
|  | R: CGAAGTGATGGTGAACGATGTGAT |  |
| *NFR* | F: ATCGTTCACCAAGAATGCGTACTG | 133 |
|  | R: CGACCAAGAACCCAAGGAACAAG |  |
| *TYR* | F: ATGAATGCCAGTGGTGAATCAGC | 129 |
|  | R: TAAAGAGGCAGCTGCTGCTTCC |  |
| *TH* | F: TCCAAATGCAAGACTCGAGGTTTG | 201 |
|  | R: TGCATACTTAGTGGTGCTGCTTGGT |  |
| *OAR1* | F: GACGACGCAAGATCCAACGACA | 136 |
|  | R: TCCTACTGCGACGGATGTATCTGG |  |
| *nAChRa4* | F: CACCAACGTCACTAGTTGTACC | 197 |
|  | R: CTGTGGTCTTCGCATAACAAGT |  |
| *nAChRa7* | F: ACAATATGGCTTGATCCAGGAT | 122 |
|  | R: GGTGTTGCTGAACCAGTTAATG |  |
| *nAChRa9* | F: CGTCGGACGCTGTTCCACTAAT | 120 |
|  | R: CGTGATTGTCGGCGGTTCTGT |  |
| *nAChRb1* | F: AGTGGAATCCTGATGAGTAT | 75 |
|  | R: CTATATCTGGAAGCCAAAGA |  |
| *ORco* | F: ACAAAGAATCAAGGACTTGTGTCCG | Kang et al., 2017b |
|  | R: ACCAGTCCAACAAAGTGTTGACAGT |  |
| *OR28* | F: TGCTCAATTTTTTAACTCTCTGGCA |  |
|  | R: CAATGCACCACCATATGACAAACT |  |
| *GR1* | F: CAGATTCTTTTCATTTTCCACCAAG |  |
|  | R: ATCCACATTTGTGAACGGCAA |  |
| *IR8a.1* | F: AGAAAGTTTTCAGAAAGTGTGCAGC |  |
|  | R: CCGTTCTGAAGAGGATTTCCATTTT |  |
| *IR8a.2* | F: CACCATAAGTAAACCATGCACATTG |  |
|  | R: AGAAAGTTTTCAGAAAGTGTGCAGC |  |
| *SOD1* | F: TTTGAACGGTGAAGATGTCAAGGG | Kang et al., 2017a |
|  | R: GTGGACCAGAGCTCATACAACCATT |  |
| *SOD2* | F: TAGTATTGGCTTTGGCTGCAACAG |  |
|  | R: TGTGGAAACCATGTTTGCCTGG |  |
| *POD* | F: TGGGGTAGTTCATTAGAAGC |  |
|  | R: CCATTAAATAAGGATGTCGA |  |
| *GST2* | F: ATGGCCCACCATCACTTGCTT |  |
|  | R: CACGTGTTTTTGGATCCTTGGGAT |  |
| *GST5* | F: TCAACATTACCGGTCTTGGTGAACC |  |
|  | R: CGTCATCACCACCAGCAACTTTAA |  |
| *18SrRNA* | F: CTATGAGTCTGGTAATTGGAAT | Gao et al., 2017 |
|  | R: GCAACAACTTAAATATACGCTAT |  |
| *RPS27* | F: CTGATACCAATGTCCACAA |  |
|  | R: CCACCACAGAATCAACTT |  |

**REFERENCE**

Gao, X. K., Zhang, S., Luo, J. Y., Wang, C. Y., Lü, L. M., Zhang, L. J., Zhu, X. Z., Wang, L., and Cui, J. J. (2017). Identification and validation of reference genes for gene expression analysis in *Aphidius gifuensis* (Hymenoptera: Aphidiidae). *PLoS ONE* 12, e0188477. doi: 10.1371/journal.pone.0188477.

Kang, Z. W., Liu, F. H., Liu, X., Yu, W. B., Tan, X. L., Zhang, S. Z., Tian, H. G., and Liu, T. X. (2017a). The potential coordination of the heat-shock proteins and antioxidant enzyme genes of *Aphidius gifuensis* in response to thermal stress. *Front. Physiol.* 8:976. doi: 10.3389/fphys.2017.00976.

Kang, Z. W., Tian, H. G., Liu, F. H., Liu, X., Jing, X. F., and Liu, T. X. (2017b). Identification and expression analysis of chemosensory receptor genes in an aphid endoparasitoid *Aphidius gifuensis*. *Sci. Rep.* 7:3939. doi: 10.1038/s41598-017-03988-z.
