# Supplementary material for: Effect of Sublethal Doses of Imidacloprid on the Biological Performance of Aphid Endoparasitoid Aphidius gifuensis (Hymenoptera: Aphidiidae) and Influence on Its Related Gene Expression
Source: Front Physiol. 2018 Dec 11;9:1729. doi: 10.3389/fphys.2018.01729 (PMC6297876; doi:10.3389/fphys.2018.01729)
Supplement: Table S2 — RNA quality of transcriptomic samples. [file Table_2.DOCX]

**Table S2. RNA quality of transcriptomic samples.**

| Samples | Concentration (ng/ µl) | Total RNA (µg) | OD260/280 | OD260/230 | Level |
| --- | --- | --- | --- | --- | --- |
| CK1 | 128 | 6.5 | 2.242 | 2.387 | A |
| CK2 | 109.1 | 5.5 | 2.05 | 2.2372 | A |
| CK3 | 113.4 | 5.7 | 2.04 | 2.273 | A |
| IMD1 | 94 | 5.0 | 2.077 | 2.16 | A |
| IMD2 | 130.2 | 6.5 | 2.01 | 2.544 | A |
| IMD3 | 99.8 | 51 | 1.97 | 2.02 | A |
